# A novel traction technique for endoscopic submucosal dissection: “traction using a rotatable clip with a line loop”

**DOI:** 10.1055/a-2886-4482

**Published:** 2026-06-22

**Authors:** Yorinari Ochiai, Minoru Oda, Junji Tanaka, Yusuke Kawai, Hiroshi Yamato, Yugo Suzuki, Shu Hoteya

**Affiliations:** 1 Department of GastroenterologyToranomon HospitalTokyoJapan; 2 Okinaka Memorial Institute for Medical ResearchTokyoJapan

Endoscopic submucosal dissection (ESD) is widely performed for the treatment of early gastrointestinal tumors and has become safe owing to advances in endoscopic devices and techniques. Traction techniques facilitate ESD and shorten procedure time. 1​ Furthermore, meta-analyses demonstrated that traction shortens the procedure time, increases the R0 resection rate, and may reduce the risk of perforation. 2​

We previously proposed a novel closure technique—“closure on traction using a rotatable clip with a line loop (CONTROLL)”—for treating mucosal defects after ESD. 3​ This technique uses a rotatable clip with a line loop ( Fig. 1 ). The loop is grasped with another clip, or the clip is passed through the loop; rotating the clip wraps the line around it, allowing length adjustment.

**Fig. 1:**
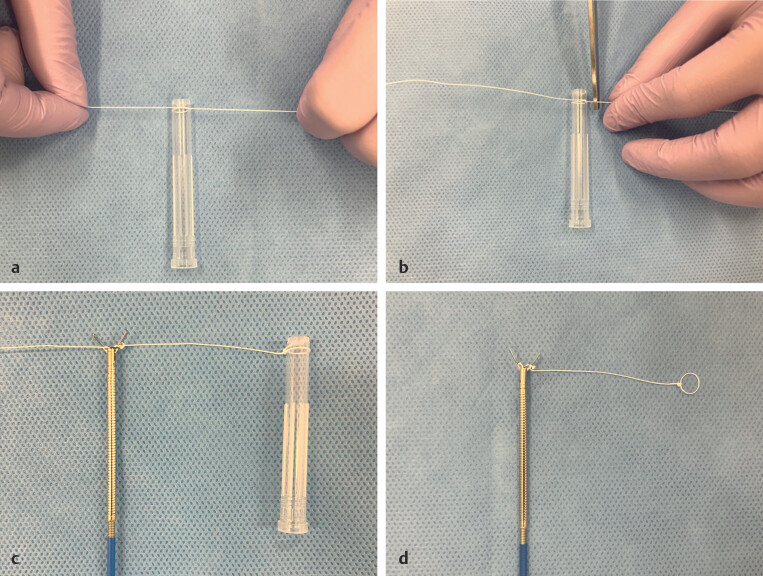
An example of how to make a clip with a line loop. ( **a** ) The line is wrapped tightly around the plastic cap of the syringe needle and securely tied, resulting in a loop approximately 5 mm in size. ( **b** ) Cut off the excess line on one side. ( **c** ) Fix the other end of the line to the clip at the intended length. A SureClip (Micro-Tech Co., Ltd, Nanjing, China) and a polyester suture (Shirakawa Co., Ltd, Tokyo, Japan) were used, and the length of the line was approximately 5 cm in this case. ( **d** ) Completion of a clip with a line loop after trimming the excess line.

Based on this, we developed a new traction method, “traction using a rotatable clip with a line loop (TRAROLL),” which widens the submucosal dissection plane, provides clearer visualization, and facilitates ESD entirely within the lumen without scope reinsertion ( Fig. 2 ).

**Fig. 2:**
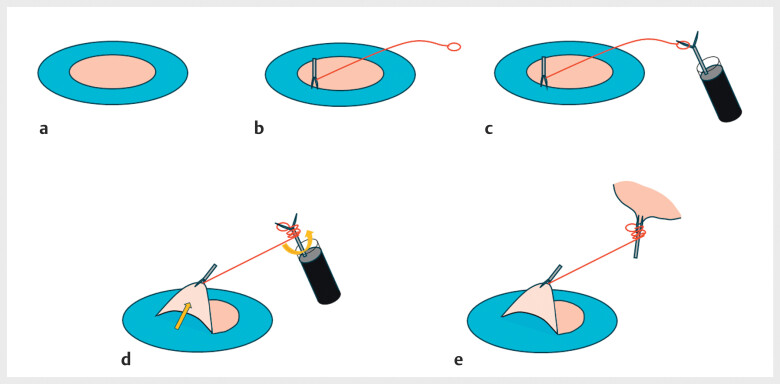
Schematic of the traction using a rotatable clip with a line loop (TRAROLL) technique. ( **a** ) Circumferential incision with creation of a mucosal flap during ESD. ( **b** ) The first clip with a line loop is placed on the mucosal flap. ( **c** ) The second clip grasped the loop and advanced in the intended traction direction. ( **d** ) The second clip is opened and rotated to wrap the line around the clip, allowing the adjustment of the line length by shortening it while observing the specimen traction. ( **e** ) After the final adjustment of the line length, the clip was deployed on the gastric wall to complete traction.

A 53-year-old woman underwent ESD for a subepithelial lesion on the posterior side of the greater curvature of the gastric fornix. After circumferential incision, the first clip (SureClip; Micro-Tech Co., Ltd, Nanjing, China) with a line loop (polyester suture; Shirakawa Co., Ltd, Tokyo, Japan) was placed on the specimen flap (line length: 5 cm). A second clip (SureClip) grasping the loop was moved to the opposite gastric wall and rotated to wrap the line around the clip, thereby shortening the line. With sufficient insufflation, the line was shortened while visually confirming traction, enabling optimal distance adjustment between the specimen and the opposite gastric wall. ESD was safely completed under effective traction ( Fig. 3 and Video 1 ). After resection, the remaining clip with the line was grasped using forceps and removed along with the specimen.

**Fig. 3:**
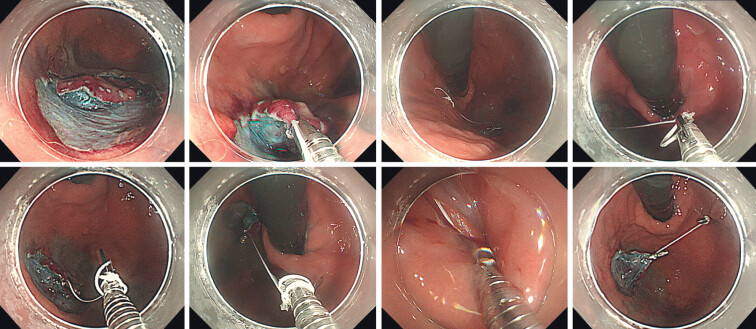
Endoscopic images of traction using a rotatable clip with a line loop (TRAROLL) technique during ESD.

**Video 1** A case of traction using a rotatable clip with a line loop (TRAROLL) technique during ESD.


TRAROLL is a simple and useful technique that provides firm and effective traction.

Endoscopy_UCTN_Code_TTT_1AO_2AG_3AD

## Ethics Approval

Written informed consent was obtained from the patient for the publication of this case report and all accompanying images.
